# Monkeypox (Mpox) virus isolation and ultrastructural characterisation from a Brazilian human sample case

**DOI:** 10.1590/0074-02760230090

**Published:** 2023-08-28

**Authors:** Milene Dias Miranda, Gabriela Cardoso Caldas, Vivian Neuza Ferreira, Ortrud Monika Barth, Aline de Paula Dias da Silva, Mayara Secco Torres Silva, Beatriz Grinsztejn, Valdiléa Gonçalves Veloso, Thiago Moreno Souza, Edson Elias da Silva, Debora Ferreira Barreto-Vieira

**Affiliations:** 1 Fundação Oswaldo Cruz-Fiocruz, Instituto Oswaldo Cruz, Laboratório de Morfologia e Morfogênese Viral, Rio de Janeiro, RJ, Brasil; 2 Fundação Oswaldo Cruz-Fiocruz, Instituto Oswaldo Cruz, Laboratório de Patologia, Rio de Janeiro, RJ, Brasil; 3 Fundação Oswaldo Cruz-Fiocruz, Centro de Desenvolvimento Tecnológico em Saúde, Instituto Nacional de Ciência e Tecnologia de Gestão da Inovação em Doenças Negligenciadas, Rio de Janeiro, RJ, Brasil; 4 Fundação Oswaldo Cruz-Fiocruz, Instituto Nacional de Infectologia Evandro Chagas, Rio de Janeiro, RJ, Brasil; 5 Fundação Oswaldo Cruz-Fiocruz, Instituto Oswaldo Cruz, Laboratório de Vírus Respiratórios, Exantemáticos, Enterovírus e Emergências Virais, Rio de Janeiro, RJ, Brasil

**Keywords:** Mpox, monkeypox virus isolation, cell culture, transmission electron microscopy (TEM), ultrastructural studies

## Abstract

**BACKGROUND:**

According to the last 2023 Monkeypox (Mpox) Outbreak Global Map from the Centres for Disease Control and Prevention (CDC), more than 100 countries with no Mpox infection report cases. Brazil stands out in this group and is the second country with the highest number of cases in the last outbreak.

**OBJECTIVE:**

To contribute to knowledge of the virus infection effects in a cellular model, which is important for diagnosis infections not yet included in a provider´s differential diagnosis and for developing viral inhibition strategies.

**METHODS:**

We describe a virus isolation protocol for a human clinical sample from a patient from Brazil, the viral growth in a cell model through plaque forming units (PFU) assay, reverse transcriptase polymerase chain reaction (RT-PCR) and transmission electron microscopy (TEM).

**FINDINGS:**

We follow the viral isolation in Vero cell culture from a Mpox positive clinically diagnosed sample and show the infection effects on cellular structures using a TEM.

**MAIN CONCLUSIONS:**

Understanding the impact of viral growth on cellular structures and its replication kinetics may offer better strategies for the development of new drugs with antiviral properties.

Monkeypox virus (MPXV) is a double-stranded DNA virus belonging to the family Poxviridae, genus Orthopoxvirus. Poxviruses have a brick- or oval-shaped structure measuring 200-400 nm.^1^ ^,^ ^2^ All poxviruses complete their replication cycle inside the infected cell’s cytoplasm. Due to the larger size of these viruses, makes it harder for viruses such as monkeypox to breach host defenses by passing through gap junctions. The larger size of the virus also makes it difficult for the virus to replicate rapidly and orthopoxviruses need a more comprehensive strategy to survive within the host. In the face of a hostile environment, poxviruses have developed an array of molecules that are encoded by virulence genes and designed to directly subvert the defenses mounted by the host.^3^ ^,^ ^4^ ^,^ ^5^ ^,^ ^6^ Poxviruses have a very broad range of eukaryotic hosts, including mammals, birds, reptiles, and insects.^7^ The major mammals hosts of Poxviruses are rodents, rabbits, and non-human primates, which can occasionally be transmitted to humans, facilitating the occurrence of human-to-human transmission.^3^ Initially isolated in cynomolgus monkeys in Copenhagen in 1958, MPXV causes a human disease is currently named as Mpox (formerly monkeypox).^8^ ^,^ ^9^

The first Mpox case in humans was reported in 1970 when the virus was isolated from a 9-month-old infant with suspected smallpox in a rural area of the Democratic Republic of Congo, following a potential zoonotic exposure.^10^ Afterward, human-to-human Mpox transmission was identified, generally occurring through droplet exposure via large exhaled droplets or by direct contact with infected skin lesions or contagious materials, with an incubation period of 6 to 13 days (5 to 21 days).^11^ The disease has a similar clinical presentation as smallpox but is less severe, presenting as a maculopapular rash, frequently on the palms and soles.^12^ The most important distinguishing sign is lymphadenopathy with swollen lymph nodes, an early Mpox symptom.^13^ ^,^ ^14^ ^,^ ^15^ ^,^ ^16^ From the 1970s to 2021, Mpox have been considered endemic in selected African countries, with sporadic outbreaks identified in other continents, mainly associated with travellers returning from endemic regions.^17^ ^,^ ^18^

On May 4, 2022, a patient with an unexplained rash and recent travel to Nigeria was admitted to an United Kingdom (UK) hospital. On May 6, 2022, MPXV was laboratory confirmed by reverse transcriptase polymerase chain reaction (RT-PCR) testing of a vesicular swab conducted by the UK Health Security Agency (UKHSA) Rare and Imported Pathogens Laboratory.^19^ Following this diagnosis, several other patients with no travel history to endemic countries were identified with Mpox in the UK and other countries from Europe and the Americas. Most of them were cisgender men, who presented with genital rash, genital oedema and proctitis.^18^ ^,^ ^20^ ^,^ ^21^ ^,^ ^22^ The situation quickly evolved into a multi-national outbreak, resulting in the declaration of Mpox as a public health emergency of international concern in July 2022.^23^

Further phylogenetic analysis pointed to a new MPXV subclade involved in the 2022 Mpox multi-national outbreak and clade II was divided into subclades IIa and IIb, with the latter associated with current cases.^24^ Mutations and natural selection have increased the transmissibility of MPXV. Recently, it has been indicated that three positive amino acid substitutions (T/A426V in MPXVgp010, A423D in MPXVgp012, and S105L in MPXVgp191), which appeared in 2019 or 2022, would be crucial for the eventual virus adaptation to humans in the current outbreak.^25^ From May 2022 to July 21 2023, more than 88,540 cases and 152 deaths were reported, most of them (97.98% of cases and 86.84% of deaths) in locations that have not historically reported Mpox.^26^ The United States of America (USA) leads the ranking with more than 30,600 cases, followed by Brazil with more than 10,900 cases.^26^ ^,^ ^27^ Since May 2022, the number of cases diagnosed outside Africa has surpassed the total number of cases detected outside the continent since 1970.^26^ ^,^ ^28^ The increasing number and rapid spread of the cases have raised concerns that MPXV may fill the ecological niche once occupied by the smallpox virus. The combined effects of deforestation, population growth, and destruction of natural reservoir habitats have recently heightened this concern.^29^ ^,^ ^30^ On May 11 2023, WHO declares global Monkeypox emergence over, but the virus is still around and further waves and outbreaks could continue.^31^

Multiple USA federal agencies, including the Administration for Strategic Preparedness and Response (ASPR), US Food and Drug Administration (FDA), National Institutes of Health (NIH), and the Centres for Disease Control and Prevention (CDC) are coordinating efforts to implement a vaccination strategy with JYNNEOS vaccine to prevent MPXV infection by demonstrating an immune response of persons considered to be at high risk for infection.^32^ The JYNNEOS vaccine is licensed as a series of two doses administered with a 28 days (four weeks) interval.^33^ In addition to this, another vaccine (ACAM2000) is licensed as a single dose for immunisation against smallpox and made available for use against Mpox under an Expanded Access Investigational New Drug (EA-IND) protocol.^32^ ^,^ ^34^ Although no specific antiviral Mpox treatment is available, tecovirimat and brincidofovir have been indicated for treatment.^35^ ^,^ ^36^ ^,^ ^37^ ^,^ ^38^ However, these drugs were approved for the management of smallpox based on animal models.^35^ ^,^ ^39^ Dose studies for these drugs have been conducted in humans, but the efficacy of these agents has not been thoroughly defined, and their use remains under monitoring.^35^ ^,^ ^40^ For example, brincidofovir may cause an increase in serum transaminases and serum bilirubin.^35^ Therefore, the development of vaccines and antiviral molecules for Mpox treatment and prevention remains a pivotal strategy to control and combat the disease.

Viral isolation in a biologically safe place and the performance of *in vitro* studies are extremely important for a better understanding of virus-cell interaction and mechanisms of infection.^41^ This knowledge is strategic to antiviral targets identification for new molecules development and drugs repurposing. Herein, we describe a virus isolation protocol for a human clinical sample from a patient from Brazil, the viral growth in a cell model through plaque forming units (PFU) assay, RT-PCR and transmission electron microscopy (TEM).

## MATERIALS AND METHODS

*Human sample and clinical description* - In the present study, we used one sample from an observational cohort study that enrolled patients with suspected Mpox virus infection between June 12 and August 19, 2022, in Rio de Janeiro, Brazil.^42^ The selected patient was a cisgender man with no comorbidities who was hospitalised in July 2022 due to severe proctitis requiring intravenous analgesia. The clinical characterisation of this patient has been previously described.^42^ He presented with fever, adenomegaly, headache, asthenia, and disseminated mucocutaneous lesions initiated six days before the medical assessment. He had sexual contact with two confirmed Mpox cases, and none of them reported travel history, pointing to a community transmission. MPXV was detected in swabs collected from penile and buttock lesions an oropharyngeal swab. These samples were received by the National Reference Laboratory in Enteroviruses and Reference Laboratory Diagnosis in MPXV (Laboratório de Enterovírus, Instituto Oswaldo Cruz, Fiocruz, Rio de Janeiro, Brazil). The virus DNA was detected by a specific MPXV qPCR protocol in an AB 7500 Real-time PCR system (Applied Biosystems).^43^ The study was conducted according to the guidelines of the Declaration of Helsinki and approved by the Ethics Review Board at Instituto Nacional de Infectologia (INI)-Fiocruz CAAE # 61290422.0.0000.5262.

*MPXV isolation* - Virus isolation was performed in the sample from the oropharyngeal swab. For virus isolation, *Cercopithecus aethiops* monkey kidney cells (Vero, BCRJ code: 0245) in a 12-well plate (2.0 x 10^5^ cells/well) were incubated with a 150 µL/well of an MPXV positive sample at two different dilutions (1:10 and 1:100) in Dulbecco’s Modified Eagle Medium (DMEM - high glucose with sodium pyruvate; Life Technologies, Grand Island, NY, USA) supplemented with 100 U/mL penicillin, 100 mg/mL streptomycin (Sigma-Aldrich, Burlington, MA, USA), and maintained at 37ºC in 5% CO_2_ atmosphere. After a 1-h incubation period, 850 µL/well of a new medium supplemented with 2% Foetal Bovine Serum (FBS; Life Technologies, South American, Brazil) was added. Cell monolayers were inspected daily under photonic microscopy for development of cytopathic effect (CPE), until six days post-infection. Then, supernatants were harvested for virus titration and genome detection, and the monolayers were collected for ultrastructural analysis by TEM. The access to the genetic heritage was approved by the Sistema Nacional de Gestão do Patrimônio Genético e do Conhecimento Tradicional Associado (SisGen; approval number: A19C586). All procedures were performed at a biosafety level 3 laboratory (BSL3), according to WHO biosafety guidelines and Biosafety in Microbiological and Biomedical Laboratories 6th Edition (CDC/NIH).^44^

*Virus titration* - Titration was performed using a 50% Tissue Culture Infectious Dose (TCID_50_) method. Monolayers of Vero cells (1,5 x 10^4^ cells/well) were incubated with 50 µL of infected cell culture supernatants, formerly starting for virus isolation, in serial 10-fold dilutions (10^2^ - 10^5^) with 10 replicates. After 1 h at 37ºC in 5% CO_2_, 50 µL of medium (DMEM high glucose supplemented with sodium pyruvate, 100 U/mL penicillin, 100 mg/mL streptomycin and 2% FBS) was added. After 72 h of incubation, the CPE was analysed by photonic microscopy and 100 µL of 4% formalin was added to fix the cells. After 3 h, this solution was harvested, and the cell monolayers were stained with 0.04% solution of crystal violet in 20% ethanol for 1 h, as described before.^45^ ^,^ ^46^ Then, the wells containing virus plaques units were counted and virus titers determined by TCID_50/mL_, according to the Reed and Muench method.^47^

*Virus genome detection* - After MPXV isolation, the virus genome was detected to confirm viral growth in cell culture. Total viral DNA was extracted using ReliaPrep^™^ Viral TNA Miniprep (Promega^®^), according to the manufacturer’s instructions. An MPXV generic real-time PCR (qPCR) test was performed according to the Centre for Disease Control and Prevention (CDC) protocol, using primers and probes already described.^43^ ^,^ ^48^ The reactions were performed using a GoTaq^®^ Probe qPCR RT-qPCR System (Promega, Madison, WS, USA) in a QIAquant 96 5plex Real-Time PCR System (Qiagen^®^).

*Ultrastructural analysis* - MPXV infected and non-infected Vero cell monolayers (control/mock) were incubated with 200 µL of trypsin-EDTA 0.25% (Life Technologies, Grand Island, NY, USA) per well and collected after six days post-infection. Then, FBS (200 µL/well) was added, and cells were fixated in 2.5% glutaraldehyde in sodium cacodylate buffer (0.2 M, pH 7.2), post-fixated in 1% osmium tetroxide in water, dehydrated in acetone, embedded in epoxy resin, and polymerised at 60ºC for three days.^49^ ^,^ ^50^ ^,^ ^51^ Ultrathin sections (50-70 nm) were obtained from the resin blocks. The sections were picked up using copper grids (uncoated 300 mesh grids) and observed using a Hitachi HT 7800 (Hitachi, Tokyo, Japan) transmission electron microscope. No staining was performed.

## RESULTS AND DISCUSSION

The first MPXV isolation and identification was performed in 1959 when monkeys were shipped from Singapore to Denmark. However, only in the 1970’s the first human infection case was confirmed in a child from the Democratic Republic of Congo, suspected to have smallpox.^52^ ^,^ ^53^ Herein, we analysed the MPXV isolation and growth starting from a positive sample from the Brazilian outbreak.

**Fig. 1:**
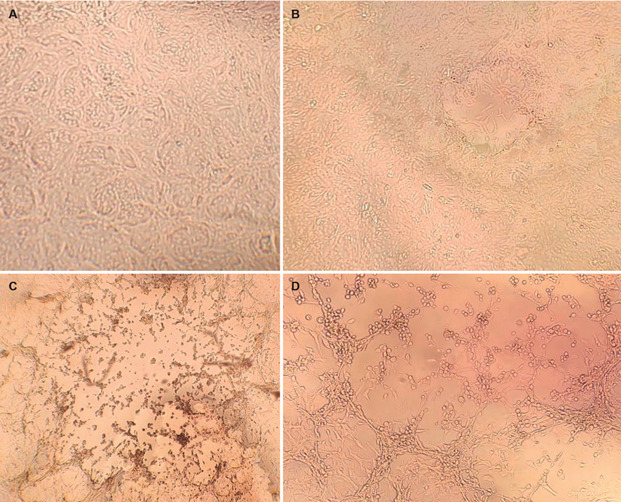
Monkeypox (Mpox) cytophatic effect (CPE) at three- and six-days post-infection. Images of Vero cells without virus inoculation (A) and inoculated with a 1:10 diluted positive sample were obtained at three days post-infection at 100x (B), and six days post-infection at 100x (C); and 200x (D). Images were obtained through an inverted microscope, EVOS XL Core Imaging System (AMEX1200, Thermo Fisher Scientific).

Viral replication in the MPXV-infected Vero cell culture was monitored daily under optical microscopy for six days. Vero cells without virus infection were used as control (Fig. 1A). After three days post-inoculation, the onset of CPE was observed in the cell monolayer infected with a 1:10 sample dilution (Fig. 1B). The CPE observed was typical as of other poxviruses, characterised by rounding of cells, plaque formation, and detachment of cells from the plastic well.^54^ ^,^ ^55^ ^,^ ^56^ ^,^ ^57^ Viral growth was maintained for another three days, when about 80% of the cell monolayer was disrupted (Fig. 1C-D). In agreement with our results, previous studies have shown MPXV isolation from clinical samples in monkey kidney cell lines (Vero E6, RMK, BGM, MA-104, LLCMK-2, OMK and BSC-40) with the onset of CPE occurring two- and three-days post infection.^58^ ^,^ ^59^ ^,^ ^60^ ^,^ ^61^ Furthermore, human lung cell line types (A549 and MRC-5), also used for MPXV isolation, showed the emergence of CPE in the same period, regardless of the cell type.^58^ ^,^ ^59^ ^,^ ^60^ ^,^ ^61^

The supernatant of the Vero cell monolayers was harvested after six days of infection. The viral growth obtained from the 1:10 dilution was titrated by TCID_50/mL_ and the viral genome detected by qPCR. The virus title was 10^4.7^ TCID_50/mL_ with a cycle threshold (Ct) equal to 30. The cell monolayers infected with the two dilutions (1:10 and 1:100) were pooled for ultrastructural analysis.

**Fig. 2:**
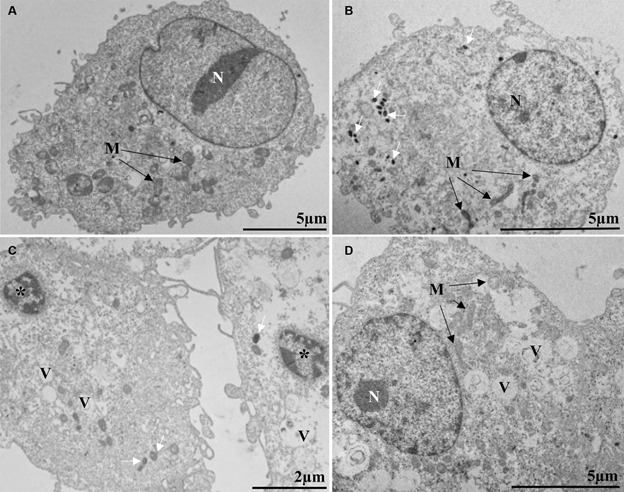
Monkeypox virus (MPXV) particles inside Vero cells six days post-infection. MPXV infected cell (A) with MPXV particles (white arrow) in cytosol (B-C), microscope images show pyknotic nuclei (*) (C), nuclei presenting an altered chromatin profile (N) and mitochondria alteration (M) (A, B, D), and numerous vesicles (V) (C-D). Images were obtained using a Hitachi HT 7800 (Hitachi, Tokyo, Japan) transmission electron microscope (TEM).

**Fig. 3:**
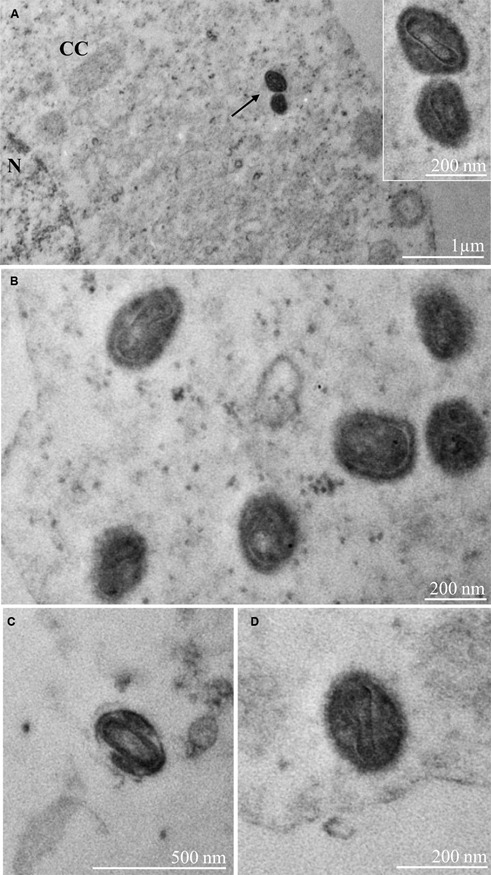
Vero cells six days post-infection (A-D) with Monkeypox virus (MPXV) particles (arrow) inside the cytoplasm (C). Virus particles presenting 391 rectangular shapes with lateral bodies and a central core. Virus particles measuring between 250 and 300 nanometers were observed. 392 nucleus (N). images were obtained using a Hitachi HT 7800 (Hitachi, Tokyo, Japan) transmission electron microscope (TEM).

Ultrastructural analysis of Vero cells on the sixth-day post-infection revealed cellular changes associated with MPXV infection, including electron-dense ribosomes, mitochondrial swelling and vacuolation, numerous vesicles, pyknotic nuclei, and nuclei presenting altered chromatin profile (Fig. 2B-D). Such changes were not observed in uninfected cells (control) (Fig. 2A). Virus particles were observed in the cytosol with a rectangular morphology and presenting lateral bodies and a central core (Fig. 3A-D) as has been observed by Bayer-Garner in ultrastructural studies by transmission electron microscopy with cutaneous biopsy specimens from patients.^62^ ^,^ ^63^ In agreement with previous reports the virion size and morphologic characteristics observed were similar to other virus particles of the Poxviridae family (200 by 300 nanometres).^64^ ^,^ ^65^ ^,^ ^66^ ^,^ ^67^

While nucleic acid amplification testes (NAATs) have largely replaced the practice of viral culture, knowing viral growth in cell models remains an important alternative to the differential diagnosis of poxvirus, even with many advances in molecular biology, still presents specific and limited targets. Thus, a wide range of pathogens not covered by common NAATs can be detected through viral culture methods.^61^ ^,^ ^68^ Furthermore, the viral variability or genetic mutations that may contribute to false NAATs negative results are less impacted by culture growth.^69^ For different viruses infections, in situations of prolonged viral shedding, culture assays are important to determining virus infectivity and phenotypic antiviral susceptibility.^70^ ^,^ ^71^ ^,^ ^72^ In addition, understanding the impact of viral growth on cellular structures and its replication kinetics may offer better strategies for the development of new drugs with antiviral properties. Beside this, the use of *in vitro* models diminishes the use of animal models.^41^ Moreover, the appearance of MPXV CPEs has been poorly characterised from the standpoint of the practicing clinical microbiologist.^61^ Thus, the evaluation of MPXV viral growth, starting from clinical samples, and the study of cellular structures modulation by viral infection is relevant.
